# Use of social audits to examine unofficial payments in government health services: experience in South Asia, Africa, and Europe

**DOI:** 10.1186/1472-6963-11-S2-S12

**Published:** 2011-12-21

**Authors:** Sergio Paredes-Solís, Neil Andersson, Robert J Ledogar, Anne Cockcroft

**Affiliations:** 1 Centro de Investigación de Enfermedades Tropicales (CIET), Universidad Autónoma de Guerrero. Calle Pino s/n Colonia El Roble, Acapulco, México; 2 CIETinternational, 511 Avenue of the Americas, #132, New York, NY 10011, USA; 3 CIET Trust Botswana, PO Box 1240, Gaborone, Botswana

## Abstract

**Background:**

Unofficial payments in health services around the world are widespread and as varied as the health systems in which they occur. We reviewed the main lessons from social audits of petty corruption in health services in South Asia (Bangladesh, Pakistan), Africa (Uganda and South Africa) and Europe (Baltic States).

**Methods:**

The social audits varied in purpose and scope. All covered representative sample communities and involved household interviews, focus group discussions, institutional reviews of health facilities, interviews with service providers and discussions with health authorities. Most audits questioned households about views on health services, perceived corruption in the services, and use of government and other health services. Questions to service users asked about making official and unofficial payments, amounts paid, service delivery indicators, and satisfaction with the service.

**Results:**

Contextual differences between the countries affected the forms of petty corruption and factors related to it. Most households in all countries held negative views about government health services and many perceived these services as corrupt. There was little evidence that better off service users were more likely to make an unofficial payment, or that making such a payment was associated with better or quicker service; those who paid unofficially to health care workers were not more satisfied with the service. In South Asia, where we conducted repeated social audits, only a minority of households chose to use government health services and their use declined over time in favour of other providers. Focus groups indicated that reasons for avoiding government health services included the need to pay for supposedly free services and the non-availability of medicines in facilities, often perceived as due to diversion of the supplied medicines.

**Conclusions:**

Unofficial expenses for medical care represent a disproportionate cost for vulnerable families; the very people who need to make use of supposedly free government services, and are a barrier to the use of these services. Patient dissatisfaction due to petty corruption may contribute to abandonment of government health services. The social audits informed plans for tackling corruption in health services.

## Background

Unofficial payments are a recognised problem in government health services, especially in developing countries [1]. These payments in cash or kind to health workers or to institutions cover items already covered by the health system [2,3]. The corrupt individual is one who possesses power, and uses that power for personal gain, financial or otherwise [4]. Unofficial payments in health services may be fostered by asymmetry of information between physicians and patients [5], faulty implementation of health care reforms [6], low pay of employees [7,8], high demand for service [7], legal vacuums [6,9], lack of information on services [10], and desire to please the doctor [11]. Despite the relatively small sums of each transaction, in total such petty corruption represents a large financial burden and a serious challenge to provision of services, especially to the poorest in the population [12].

While many studies have reported on how common unofficial payments are or their significance [13,14], there is little epidemiological analysis of the risk factors for such payments. It is difficult to study unofficial payments because by their nature they are “hidden” and in many countries are actually illegal; neither party to the transaction will admit to it [15-17]. Social audit methods [18] help to study unofficial payments as seen by intended service users, incorporating the perspective of service providers, and providing pointers for context-specific actions to tackle the problem [19,20].

Drawing on the work reported in a doctoral thesis [21], we describe the main lessons from social audits of petty corruption in health services in South Asia (Bangladesh, Pakistan), Africa (Uganda and South Africa) and Europe (Baltic States).

## Methods

In Uganda and the Baltic States the social audits focused specifically on corruption in health and other services [16,22], while those in South Africa, Bangladesh, and Pakistan were studies of perceptions, use, and experience of health and other public services which included questions about unofficial payments [19,23-25]. In Bangladesh and Pakistan we undertook a series of surveys, allowing measurement of changes over time. Detailed descriptions of the sampling, data collection, and analysis are available in the reports and publications from the social audits [16,19,22-25].

Several features were common to all the social audits: interviews with household respondents in a representative sample of communities to ask about their use and experience of health services; focus groups with separate male and female groups in the sample communities to discuss key findings from the household interviews; institutional reviews to examine health facilities serving the sample communities; interviews with service providers; and discussions with service planners and policy makers. Most of the social audits included questions to all households (whether or not they had used government health services) about their views of health services, about perceived corruption in the services, and about their use of government and other health services. Questions specific to each context categorised socio-economic status and vulnerability of households. Additional questions to recent users of services asked about their experience of making payments, official and unofficial, the amounts of the payments, service delivery indicators (such as waiting time and availability of information from the services), and their satisfaction with aspects of the service they received. Table 1 gives information about the surveys and their samples.

**Table 1:** The social audits reviewed

Country	Survey(s)	Sample
Uganda	National integrity survey 1998	18,412 households in all 45 districts 1,595 public service workers 348 male and female focus groups
South Africa Gauteng province	Survey of health services performance 2003	5,490 households 120 focus group discussions Reviews of health facilities and interviews with health workers
Baltic states (Estonia, Latvia, Lithuania)	Survey of corruption in health and licensing services 2002	Total 10,320 households in the three countries 90 focus group discussions Reviews of 104 health facilities
Bangladesh	Three surveys: 1999, 2000, 2003	In each survey, some 25,000 households, 200 health facilities, 200 key informants and 500 focus groups. In 2000 and 2003, 2,000 health workers
Pakistan	Two surveys: 2002 and 2004	Households: 57,321 in 2002, 53,960 in 2004 In each survey 800 focus groups In 2004, 1,000 local government key informants

Data entry in the surveys relied on Epi Info [26] and included double data entry with validation to minimise keystroke errors. Analysis relied on Epi Info and CIETmap open-source software [27,28]. We calculated weighted frequencies of outcomes (such as making an unofficial payment), and examined variables related to key outcomes in univariate and multivariate analysis, using the Mantel-Haenszel procedure [29]. We report here associations as adjusted Odds Ratios (ORa) and 95% confidence intervals (CI).

In each country we attempted to use the findings from the social audits (including focus group discussions) to inform improvement of services. In some countries, like Uganda, the focus was specifically on corruption, while in other countries such as Pakistan and Bangladesh, it was overall service delivery. The form and extent of this follow-up activity varied between countries; it is not covered in this review but is described in the reports of the individual social audits [16,19,22-25].

## Results

### Household views about health services

In the social audits in seven countries reviewed here, most households held negative views about government health services. In the Baltic States only a minority rated government health services as good (19% in Latvia, 26% in Lithuania, and 39% in Estonia), while in Gauteng province in South Africa just 19% considered health as the best run government department. In Bangladesh, fewer households rated government health services as good in 2000 and 2003 than in 1999 (10% vs. 38%), despite a health services reform programme. In Pakistan, only about a quarter of households reported satisfaction with government health services (23% in 2002 and 27% in 2004).

At the same time, many households perceived government health services as corrupt. In the Baltic States, around half the households rated corruption in government health services as high (43% in Estonia, 45% in Latvia, and 64% in Lithuania). Some 19% of households in Gauteng province, and 27% of households in Uganda rated health as the most corrupt government service. Focus groups clarified that corruption (having to pay for supposedly free services) was a major reason for low rating of government health services.

“People are charged Rs 200 as a fee in public hospitals. How can a poor person be expected to come up with such a lot of money?” Male focus group, Pakistan.

“Health workers ask for ‘chai’ [bribes]; if you don't give it, you are not treated.” Female focus group, Uganda.

### Household use of government health services

In South Asia, only a small fraction of households chose to use government health services, in preference to services from other providers. In Pakistan, 29% of households in 2002 and 24% in 2004 reported they usually used a government health facility for medical attention, while 45% used private qualified practitioners, and a quarter used unqualified medical practitioners (24% in 2002 and 29% in 2004). In Bangladesh, use of government health services for treatment in the last month fell from 13% of households in 1999 to 10% in 2003, while the proportion using private or NGO services in the same period rose from 30% to 49%. Focus groups gave important reasons for avoiding government health services: the need to pay for supposedly free services, non-availability of medicines in facilities (usually perceived as due to diversion of the supplied medicines), and bad behaviour from health workers, especially towards poor patients.

“I went to the hospital to get medicines for stomach pain. They did not give me any medicines. They wanted money from me but I did not have money, so they did not give me medicine. If we have to buy medicines from the hospital then it is better to go directly to the pharmacy.” Female focus group, Bangladesh

“Why should we pay tax, VAT, and then “cost-sharing” in [government] hospitals?” Male focus group, Uganda

### Unofficial payments in health services

Our surveys asked about unofficial payments in government health services in slightly different ways. In Uganda and Gauteng province, we asked service users in households if they had made direct payments to health workers. In Uganda in 1998, 28% reported making such a payment, while in Gauteng province in 2003 only 1% reported this. In Bangladesh and Pakistan, we asked about a range of official and unofficial payments in government health facilities. In Bangladesh, about one in five government health service users made direct payments to health workers (21% in 1999, 20% in 2000, 18% in 2003). In Pakistan in 2004, only 5% of users of government health services reported paying directly to a service worker; however, nearly all paid something for the visit, including 65% who paid for medicines outside the facility and 5% who paid for medicines inside the facility. Three quarters paid for a registration ticket, and one half paid more than the official rate for this ticket. In the Baltic States, a small proportion of government health service users reported they made an unofficial payment, usually to the doctor (1% in Estonia, 3% in Latvia, 8% in Lithuania).

There were differences between countries. In the Baltic States, more service users reported giving a “gift” to a health worker than making an unofficial payment: about 14% in all three countries reported giving a gift. In the Baltic States, service users initiated most (around 80%) of the reported unofficial payments; this contrasts with Uganda, where health workers requested or demanded 90% of the unofficial payments. In South Asia and Africa, focus group participants expressed anger about being forced to make unofficial payments. In the Baltic States, however, only one half of households considered unofficial payments to health workers as a form of corruption, and focus groups suggested factors such as timing, amount, intention, and who initiated the payment determined whether it should be considered corruption.

### Factors related to making unofficial payments

Separate multivariate analyses for each country examined variables potentially associated with making unofficial payments. Only one of our social audits produced any evidence linking household economic status (assessed according to the context of each country) with making an unofficial payment. In Lithuania, households reporting an income sufficient for their needs were more likely to have made an unofficial payment (ORa 1.53, 95% CI 1.04 – 2.14). However, in Latvia (ORa 2.53, 95% CI 1.69 – 3.80) and Lithuania (ORa 2.10, 95% CI 1.55 – 2.86) respondents with more education, and in Bangladesh people from households with a literate head (ORa 1.48, 95% CI 1.18 – 1.85), were more likely to report making an unofficial payment. In Uganda, people from households with a male head (likely to be less vulnerable than households with a female head) were more likely to report an unofficial payment (ORa 1.24, 95% CI 1.10 – 1.40). In the Baltic States, gift giving was strongly associated with making unofficial payments. There was little evidence that making an unofficial payment was associated with getting a better or quicker service. Indeed, in Gauteng, South Africa, those people who waited less than 30 minutes for medical attention were *less* likely to report making an unofficial payment (ORa 0.48, 95% CI 0.23 – 1.00). In Uganda, users of health services, as well as other government services, who paid a bribe took longer to complete their dealings with the service (ORa 2.04, 95% CI 1.89-2.22), and saw more individual staff members.

### Unofficial payments and satisfaction with service received

Those who paid unofficially to health care workers were not more satisfied with the service. In general, there was no association between making an unofficial payment and reported satisfaction with the service received. In Lithuania (ORa 0.49, 95% CI 0.33 – 0.73) and Uganda (ORa 0.27, 95% CI 0.24 – 0.29), people who made an unofficial payment were less likely to report satisfaction with the service they received, taking other factors into account.

## Discussion

Several points in the health care process are vulnerable to corruption, including the point of service delivery [30]. Measuring corruption at different levels of health care requires different techniques. There are numerous opportunities for corrupt practices in hospital systems, including bulk purchases of medicines and supplies, that do not directly involve service users, that might be detected by financial audits and stock-taking. Social audit is a powerful tool for detecting and measuring corruption at the point of service delivery. The approach can help to find factors that increase the risk of corruption, as well as perceptions of the public and service providers.

Hirschman's theory of exit, voice and loyalty [31] has sometimes been applied in health services research [32] and, more pertinently, to health services delivery in developing countries [33]. The theory suggests that giving users greater voice in the delivery of health services should help to reduce desertion rates. Our series of social audits in Bangladesh and Pakistan suggested that the public were continuing to abandon government health services to seek other health care options. Quantitative and qualitative evidence from these social audits suggested that the experience and perception of corruption in the services was a factor driving people away. Other authors have suggested that pressure by health employees for payments may function as a barrier to using public health services [34].

Unofficial payments have different forms and connotations depending on the context. Giving gifts to health workers seems normal and distinct from unofficial payments in the Baltic States, as in other countries in the former Soviet Union [35]. There is no such tradition of gift giving in Africa and South Asia. In the Baltic States, service users initiated most of the payments. The trigger for this might be desire for a better quality service [36], or based on their own experience or that of their social network [37]. Nevertheless, the intention to buy a better service was apparently thwarted, since the service users who made an unofficial payment were not more satisfied with the service they received. In Uganda, almost all the reported unofficial payments were in response to a demand from the service provider, and those who paid apparently experienced a slower service. This suggests extortion by the more powerful service provider. Focus group participants in Uganda, Pakistan and Bangladesh said that people felt trapped, and forced into making unofficial payments.

Lithuania excepted, the social audits presented here did not find better-off users of government health services were more likely to make unofficial payments. In relation to their smaller overall incomes, unofficial expenses for medical care represent a disproportionate cost for vulnerable families, the very people who need to use supposedly free government services, and thus are a barrier to the use of these services.

## Conclusions

Social audits allowed measurement of the frequency of unofficial payments, the factors associated with them, and what they meant to service users. The findings of these social audits contributed to the understanding of unofficial payments in government health services in the countries concerned. Official bodies tasked with reducing corruption took account of the findings when formulating plans to tackle the problem of corruption in these services.

## Competing interests

The authors declare they have no competing interests.

## Authors' contributions

SPS supported the social audit in the Baltic States and South Africa, undertook analysis, and led the drafting of this article.

NA provided technical oversight for all the social audits and supported drafting of this article.

RJL supported the drafting of this article.

AC led the social audits and their analysis in Uganda, Bangladesh and the Baltic States and supported the drafting of this article.
